# Primary Health Care approach to managing chronic kidney disease

**DOI:** 10.4102/safp.v68i1.6276

**Published:** 2026-06-03

**Authors:** Ramprakash Kaswa, Swati Meel

**Affiliations:** 1 Department of Family Medicine and Rural Health, Faculty of Health Sciences, Walter Sisulu University, Mthatha, South Africa; 2 Department of Health, Mthatha Regional Hospital, Mthatha, Eastern Cape, South Africa; 3 Netcare Greenacres Hospital, Gqeberha, Eastern Cape, South Africa

**Keywords:** CKD, diabetes, hypertension, Primary Health Care, primary care providers

## Abstract

Chronic kidney disease (CKD) is a growing global health issue among ageing populations, driven by increasing diabetes and hypertension. Managing advanced stages of CKD with dialysis or transplants is costly. Early stages often have no symptoms, making early detection important for prevention. Primary care providers can help identify and manage CKD early by performing annual screening for high-risk patients, including blood pressure checks, urine tests for albuminuria or proteinuria and estimating glomerular filtration rate from serum creatinine. For those with CKD, renal protective measures are essential to slow disease progression. This narrative review highlights the critical role of primary care providers in the comprehensive and integrated CKD management.

## Introduction

Chronic kidney disease (CKD) is an imminent global health issue. According to the Global Burden of Disease Study 2023, more than 850 million people are affected by kidney disease, twice as many as those living with diabetes and 20 times more than those with human immunodeficiency virus or acquired immunodeficiency syndrome. Worldwide, CKD has shown steady growth, climbing from the 27th leading cause of death in 1990 to the 9th place in 2023.^1^ This has prompted the World Health Organization kidney health resolution to prioritise kidney disease within the non-communicable disease programme.^2^ The rising prevalence is associated with ageing populations and an increasing burden of risk factors from non-communicable diseases such as diabetes and hypertension, which together are responsible for over half of CKD-related deaths.^3,4^

Diabetes is the primary cause of CKD, including end-stage kidney disease (ESKD). The prevalence of type-2 diabetes among adults continues to increase, mainly as a result of population ageing and the obesity epidemic.^5^ Additionally, an ageing population is a risk factor for CKD, partly because normal ageing-related physiological changes decrease glomerular filtration rate (GFR), especially after age 50. However, there is ongoing debate in the literature about whether to use absolute or age-adjusted cut-off values to detect CKD and assess risk. Because GFR declines with age, a fixed threshold rate can lead to underdiagnosis of CKD in young adults and overdiagnosis among the elderly.^6^

Chronic kidney disease is the seventh leading cause of cardiovascular mortality. Even a moderate decline in kidney function is associated with a significantly increased risk of cardiovascular events and death. While those progressing to ESKD require specialised care, such as transplantation, dialysis or sometimes palliation, these costs have a substantial impact on the healthcare system.^7^ In South Africa, there are approximately two nephrologists per million people, which is below the global standard and inadequate for CKD management.^1^

Epidemiological data from South Africa emphasise the importance of prioritising kidney disease on the public health agenda.^2^ The prevalence rates for CKD in South Africa ranged from 6.4% to 8.7%, in sub-Saharan Africa 10.7% – 13.9%, in Africa 4.6% – 10.1% and globally 4.1% – 10.6%. Notably, the burden of CKD is most significant among historically marginalised populations, which often have limited access to optimal management of kidney conditions. This substantially contributes to existing socioeconomic disparities in health outcomes.^7^

Despite the rising burden of CKD, there is no consensus on population- or targeted-screening. Early detection and management in primary care are vital for preventing CKD, delaying progression and avoiding adverse outcomes. An effective strategy involves implementing the 78th World Health Assembly resolution on the prevention and control of kidney diseases,^2^ including ongoing training, and addressing the shortage of human and healthcare resources in primary care, which are crucial for control.^7^

Primary Health Care serves as the entry point for community health needs. Most patients with risk factors or in the early stages of CKD can be managed in primary care settings.^8^ Therefore, primary care providers must identify and treat CKD. This narrative review aims to guide primary care providers on current practices for early detection and management of kidney disease in Primary Health Care. The search strategy used the keyword ‘Chronic Kidney Disease or CKD or Chronic Renal Disease and Primary care or Primary Health Care’ and involved academic databases such as PubMed, Scopus, Web of Science and Google Scholar.

## Diagnosis

Chronic kidney disease is defined by structural or functional abnormalities in the kidney that last for at least 3 months. The Kidney Disease: Improving Global Outcomes (KDIGO) defines CKD as ‘Decreased GFR < 60 mL/min per 1.73 m^2^ (GFR categories G3a–G5), Albuminuria (albumin to creatinine ratio (ACR) > 3 mg/mmol) and markers of kidney damage (one or more), such as urine sediment abnormalities, persistent haematuria, electrolyte and other abnormalities due to tubular disorders’. These abnormalities can be identified through biochemical tests, urinalysis, histological examination, radiological imaging and a patient’s history of kidney transplantation.^4^

Kidney Disease: Improving Global Outcomes recommends staging of CKD using a validated creatinine-based estimating equation to determine GFR (eGFRcr). In certain circumstances, when the accuracy of creatinine may be questionable, an estimating equation that incorporates creatinine and cystatin C is advised. These situations include patients with a small body habitus, malnutrition, low protein diet, malignancy, cirrhosis, catabolic states and steroid use.^4,6^

## Staging of chronic kidney disease

**FIGURE 1:**
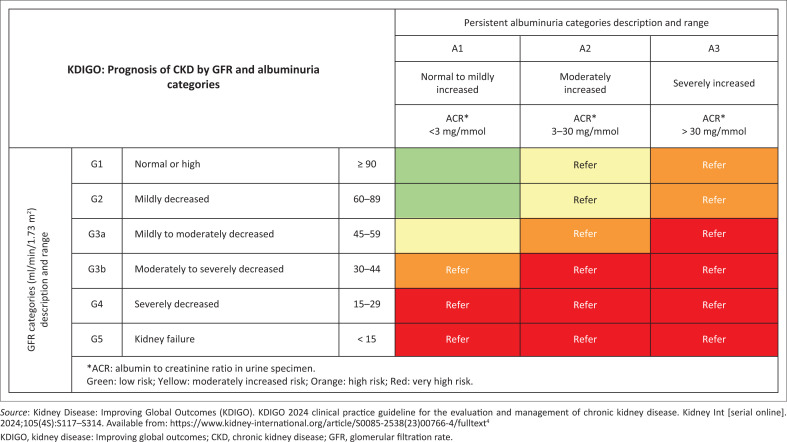
Chronic kidney disease staging based on albuminuria and glomerular filtration rate category.

Staging of CKD is decided by GFR categories (G1–G5), Albuminuria categories (A1–A3) and underlying causes. These elements are vital for assessing CKD severity and associated risks. Staging helps predict patient outcomes and informs treatment strategies. Higher albuminuria levels are associated with worse cardiovascular outcomes. The combination of CKD cause, stage and albuminuria level affects prognosis.^4,9^ Figure 1 illustrates CKD staging based on KDIGO diagnostic criteria.

### Screening

There is a notable disparity between the burden of CKD and the availability of healthcare resources. Access to kidney replacement therapy (KRT) remains limited, especially as diabetes and hypertension rates rise.^3^ Instead of population-wide screening, a targeted approach within Primary Health Care settings is practical and cost-effective. The focus should be on individuals with risk factors for CKD, detailed in Box 1. Screening methods include measuring serum creatinine to estimate GFR and conducting urine tests for albumin excretion.^10^

BOX 1 Commonly identified risk factors for chronic kidney disease.

Diabetes

Hypertension

Obesity

Age > 65 years

Family history of kidney disease

Gout

Recurrent kidney calculi

Nephrotoxins (cadmium, lead, mercury, polycyclic hydrocarbons, pesticide exposure)

HIV

Recurrent urinary tract infections

Metabolic syndrome

Cardiovascular disease

Autoimmune disease

Previous AKI

Structural urinary tract abnormalities

Prostate pathology

Gestational conditions (preterm birth, small gestational size, pre-eclampsia/eclampsia)

Chronic NSAIDs use

AKI, acute kidney injury; HIV, human immunodeficiency virus; NSAIDs, nonsteroidal anti-inflammatory drugs.

The American Diabetes Association and KDIGO recommended annual CKD screening begin with onset of type 2 diabetes because of preexisting risk. While in type 1 diabetes screening should begin five year after diagnosis. The Table 1 listed the common risk factors often needs CKD screening.^1,4^

**TABLE 1:** Common causes of chronic kidney disease.

System affected	Underlying Causes
Pre-renal causes	Cardiorenal syndrome – chronic heart failure may lead to CKD Liver cirrhosis
Renal vascular causes	Hypertensive nephrosclerosis Renal artery stenosis Athero-emboli of renal vasculature Vasculitis Fibromuscular dysplasia
Glomerular disease – characterised by proteinuria and haematuria from urine examination	Primary glomerular disease ■ Membranoproliferative glomerulonephritis ■ Ig A nephropathy ■ Focal segmental glomerulosclerosis ■ Minimal change disease
	Secondary glomerular disease ■ Lupus nephritis ■ Diabetic nephropathy ■ Rheumatoid arthritis ■ Amyloidosis ■ Light chain deposition disease ■ Malignancy
Tubulo-interstitial disease	Drug-induced e.g. NSAIDs, contrast agents Infections e.g. post-streptococcal, HIV and hepatitis B Toxins e.g. environmental and heavy metal exposures Multiple myeloma cast nephropathy Genetic disorder e.g. autosomal dominant tubule-interstitial disease
Urinary tract obstruction	Renal stone disease Benign prostatic hyperplasia

*Source*: Kidney Disease: Improving Global Outcomes (KDIGO). KDIGO 2024 clinical practice guideline for the evaluation and management of chronic kidney disease. Kidney Int [serial online]. 2024;105(4S):S117–S314. Available from: https://www.kidney-international.org/article/S0085-2538(23)00766-4/fulltext^4^

HIV, human immunodeficiency virus; CKD, chronic kidney disease; NSAIDs, nonsteroidal anti-inflammatory drugs.

### Common causes of chronic kidney disease

Causes of CKD are categorised based on the underlying pathophysiological mechanism, as shown in Table 1. These include pre-renal, renal vascular, glomerular, tubulointerstitial, hereditary and obstructive causes. An ultrasound of the kidneys and urinary tract, together with Doppler imaging, can detect urological issues, renal artery stenosis, tumours and cystic kidney diseases, and provide valuable information on kidney structure to support diagnoses of acute or chronic conditions. All suspected cases of glomerular disease require serological testing.^3,10^

### Specialist referral

Primary care physicians are essential in making timely referrals to nephrologists, enabling early intervention to slow CKD progression and prepare patients for KRT. Evidence indicates that rapid referrals improve KRT readiness, reduce dialysis catheter use, reduce emergency dialysis episodes and improve survival rates. Below is a guideline for when to refer patients promptly to a nephrologist^1,11^:

- Sudden decline in eGFR > 5 mL/min/1.73 m^2^ in 1 year.
- Despite treatment optimisation major proteinuria (urine protein > 1 g/day).
- Microscopic haematuria persists.
- Stage G4 (eGFR < 30 mL/min) who need KRT.
- Women planning for pregnancy or currently pregnant.
- Persistence hypertension despite optimum treatment.
- Isolated microscopic haematuria does not originate from urological causes.
- Hereditary CKD causes (e.g. polycystic kidney disease).
- Persistent hyperkalaemia.
- Underlying cause not known.

### Prognosis of chronic kidney disease

Patient with CKD are at risk of progression. Either a decline in GFR category or a drop in GFR category accompanied by at least a 25% decrease in eGFR from baseline defines CKD progression. For patients with CKD stage G2 or higher, eGFR should be checked twice annually. A more practical approach to monitoring CKD progression involves evaluating annual changes in eGFR. This method can assist primary care physicians in tracking eGFR over time. Recognising patients with risk factors for rapid decline early is essential, and if rapid decline is confirmed, they should be referred to a nephrologist for specialised management.^4^

## Management of chronic kidney disease

The primary objectives of CKD management are to slow disease progression, lower cardiovascular risk, identify patients needing renal replacement therapy, manage CKD-related complications and modify medications according to GFR. Table 2 presents common strategies for delaying CKD progression, while Table 3 details typical complications in CKD patients and their management.

**TABLE 2:** Common strategies for delaying the progression of chronic kidney disease.

Risk factors		Management
1	Lifestyle changes	Smoking cessation Maintain a healthy weight Physical activity compatible with cardiovascular tolerance
2	Dietary management	Low-salt diets (< 2 g/day) Low-protein diet (0.6 g/kg/day – 0.8 g/kg/day)
3	Blood glucosecontrol	Aim for HbA1c of 6.5% – 7.0% Use of sodium-glucose co-transporter-2 (SGLT2) inhibitors
4	BP and proteinuria	Target systolic BP for all adult CKD patient is < 120 mmHg
5	Management of complications	Mineral bone disease Fluid overload Cardiovascular risk Atrial fibrillation Metabolic acidosis Anaemia
6	Avoid nephrotoxicdrugs	Nonsteroidal anti-inflammatory drugs (NSAIDs) Certain antibiotics (e.g. aminoglycosides, vancomycin) ACE inhibitors and ARBs Chemotherapy drugs Antifungals e.g. amphotericin B Proton pump inhibitors Contrast dyes

*Source:* Kidney Disease: Improving Global Outcomes (KDIGO). KDIGO 2024 clinical practice guideline for the evaluation and management of chronic kidney disease. Kidney Int [serial online]. 2024;105(4S):S117–S314. Available from: https://www.kidney-international.org/article/S0085-2538(23)00766-4/fulltext^4^

BP, blood pressure; CKD, chronic kidney disease; ACE, angiotensin-converting enzyme; ARB, angiotensin II receptor blocker.

**TABLE 3:** Common complications of chronic kidney disease and their management approach.

No	Complication	Management
1	Anaemia	The haemoglobin target in CKD is 10 g/dL – 12 g/dL. Annual screening with stage G3a onwards Iron supplements Erythropoietin-stimulating agent (nephrologist referral) Blood transfusion (in emergency only to avoid sensitisation, which will impact kidney transplantation)
2	Mineral bone disease	Serum calcium, phosphate, alkaline phosphatase and parathyroid hormone (PTH) levels measured annually with stage G3a onwards Dietary phosphate restriction Low-protein diet and phosphate binders Calcium and vitamin D supplements
3	Fluid overload	Assess fluid status by recognising symptoms and signs of elevated jugular venous pressure, crackles in the lungs and pedal oedema Fluid and salt restriction Loop diuretics
4	Cardiovascular risk	BP control (target systolic blood pressure of < 130 mmHg) Glycaemic control (Targeted HbA1c of 6.5% – 7.0%) Lipid-lowering therapy e.g. statin Antiplatelet only as secondary prevention
5	Atrial fibrillation	Kidney adjusted dose of oral anticoagulant after a bleeding risk score Beta blockers (carvedilol) Cardioversion Antiarrhythmic therapy (amiodarone)
6	Metabolic acidosis	Oral alkali supplements e.g. sodium bicarbonate, sodium citrate etc Diet with a predominance of plant proteins over animal proteins, and high amounts of fruits and vegetables

*Source:* Cheo SW, Low QJ, Lim TH, et al. A practical approach to chronic kidney disease in primary care. *Malays Fam Physician*. 2022;17(1);10–19. https://doi.org/10.51866/rv1186

No., number; BP, blood pressure; CKD, chronic kidney disease.

## General measures

General lifestyle advice remains important at all stages of CKD. This includes quitting smoking, losing weight, following a low-salt diet (< 2 g/day) and avoiding nephrotoxic substances. Chronic kidney disease patients should be encouraged to participate in suitable physical activity for at least 30 min, five times a week, depending on their cardiovascular health and tolerance. Smoking is linked to faster CKD progression; quitting can help reduce the risk of worsening kidney function and cardiovascular problems. Additionally, adopting a more plant-based diet and limiting animal protein and processed foods can reduce proteinuria and metabolic acidosis. Dietary advice from a qualified dietitian supports managing sodium, phosphate, protein and potassium intake, tailored to CKD stage and comorbidities.^3,10^

For the elderly, dietary protein restriction must be individualised. Special attention is needed for frail and sarcopenic older patients to prevent malnutrition. This involves prioritising the most urgent clinical issues. According to Geriatric guidelines, a daily protein intake of 1 g/kg – 1.2 g/kg of body weight may be suitable for patients with stable CKD and other significant age-related concerns. Conversely, in an otherwise stable patient with progressing CKD, a more limited protein intake might be better. Such decisions should involve input from the patient, family and caregivers.^4,8^

### Hypertension

Optimal blood pressure management is the key intervention for delaying progression and lowering cardiovascular risk in patients with CKD. In particular, control of hypertension in over 80% of CKD patients is often inadequate. Hypertensive patients with CKD should aim for a target systolic blood pressure of < 120 mm Hg. Patients with frailty, a high risk of falls and fractures, limited life expectancy or symptomatic postural hypotension should consider less aggressive blood pressure reduction.^4,12^

An angiotensin-converting enzyme (ACE) inhibitors or an angiotensin II receptor blockers (ARBs) is the first-line treatment for people with diabetic kidney disease or significant proteinuria (> 1 g/day). These medications help decrease proteinuria and slow the progression of CKD, regardless of blood pressure control. It’s essential to monitor kidney function and serum potassium levels 2–4 weeks after starting or increasing the dose of an ACEI or ARB. Use dihydropyridine calcium channel blockers and/or diuretics if needed to meet the personalised blood pressure goals. Consider adding a mineralocorticoid receptor antagonist if hypertension is resistant, as long as the eGFR is ≥ 45.^4,6^

### Glycaemic control

The KDIGO guidelines recommend an individualised HbA1c target ranging from < 6.5% to < 8% for patients not on dialysis. As kidney disease progresses to stages G4 and G5, HbA1c readings become less dependable. In such cases, using continuous glucose monitoring systems or self-monitoring of blood glucose can help improve glycaemic control and reduce hypoglycaemic incidents. The current target for glycaemic management is an HbA1C of 6.5% – 7.0%. Achieving and maintaining this HbA1C range can lower the risk of cardiovascular disease, albuminuria, and renal function decline over time. However, the advantages of strict diabetes control need a balance against the risk of hypoglycaemia, particularly in frail patients with advanced CKD. Dosages of medications like metformin and sulphonylureas may need adjustment based on eGFR to prevent hypoglycaemia and lactic acidosis.^10^

Sodium-glucose co-transporter-2 (SGLT2) inhibitors have a protective effect on the kidneys by lowering the risk of ESKD, slowing CKD progression and reducing cardiovascular events. These inhibitors benefit CKD patients (median eGFR 43 mL/min) regardless of diabetes status. Adults with eGFR above 20 mL/min per 1.73 m^2^ and urine ACR below 20 mg/mmol should use SGLT2 inhibitors. Additionally, long-acting glucagon-like peptide-1 (GLP-1) receptor agonists are advised for adults with type 2 diabetes who have not met individualised glycaemic targets despite using metformin and an SGLT2 inhibitor, or who cannot take these medications.^4^

### Hyperkalaemia

Evaluate dietary potassium intake (dietary referral) and advise moderation as needed. Use loop diuretics appropriately, optimise serum bicarbonate levels and start potassium exchange agents. For resistant cases, consider lowering the dose or stopping mineralocorticoid receptor antagonists, nonsteroidal anti-inflammatory drugs and renin-angiotensin system inhibitors.^4,13^

### Dyslipidaemia

All patients with newly diagnosed CKD should undergo lipid profile testing to assess their cardiovascular risk. Adults > 50 years with CKD should receive statins to reduce major atherosclerotic events. For adults < 50 years with CKD not on renal replacement, lipid-lowering therapy is advised if they have diabetes and a history of coronary artery disease or ischaemic stroke. Low-dose aspirin is recommended for secondary prevention of recurrent ischaemic cardiovascular events in individuals with established risk.^4,6^

### Acute kidney injury and infection

Chronic kidney disease patients are at a high risk of developing acute kidney injury (AKI) following superimposed infection. Each episode of AKI further diminishes renal function and accelerates the progression of CKD. Recurrent infections and the use of nephrotoxic medications are well-established risk factors for AKI. Early detection of hypovolaemia, prompt antibiotic therapy for infections and regular monitoring of eGFR are essential to prevent further renal damage. Temporary discontinuation of metformin, SGLT2 inhibitors or ACEi and/or ARBs is advised in cases of dehydration or hypovolemic shock. All CKD patients should receive annual influenza and pneumococcal vaccinations after excluding contraindication.^4,14^

## Management of chronic kidney disease complications

### Chronic kidney disease and responsible prescribing of medicines

All CKD patients should be monitored to ensure the safe and effective use of pharmacological agents. The following measures are advised:

- Avoid polypharmacy.
- Identify nephrotoxic and deprescribe.
- Dose adjustment in alignment with eGFR.
- Prefer the drugs that do not eliminate through kidney.
- Educate the patient about over-the-counter medications on the kidney.

### Kidney supportive care

Kidney supportive care (KSC) is a holistic approach that aims to improve the quality of life for individuals affected by kidney disease, whether directly or indirectly. It recognises patients’ physical, psychosocial and spiritual needs. Kidney supportive care helps people manage both living and dying stages and should be available to all with advanced CKD according to their supportive care needs. The main elements of KSC include^8,11^:

- Shared decision-making.
- Symptom management.
- Kidney replacement therapy.
- Integration with community services.
- Crisis management.
- Planning for advance care.
- Culturally sensitive spiritual care.
- Palliative care.

## Conclusion

In conclusion, CKD is a common diagnosis in Primary Health Care. Recently, its prevalence has increased disproportionately among patients in primary care, driven by an ageing population and the rising burden of non-communicable diseases, especially diabetes. Primary care providers play a vital role in early detection and screening, as CKD often exhibits no symptoms in its early stages. Annual screening for blood pressure, urine dipstick tests for proteinuria or albuminuria and serum creatinine is recommended for individuals at high risk of CKD. For established CKD patients, renal protective strategies include a multidisciplinary approach with dietary management, drug stewardship, prompt infection management, optimisation of blood glucose and blood pressure and measures to reduce cardiovascular risk. As the first point of contact for community health needs, primary care providers should effectively manage most at-risk or early-stage CKD patients through an integrated approach to non-communicable disease management.
